# Significant association between renal function and amyloid-positive area in renal biopsy specimens in AL amyloidosis

**DOI:** 10.1186/1471-2369-13-118

**Published:** 2012-09-24

**Authors:** Takeshi Kuroda, Naohito Tanabe, Daisuke Kobayashi, Yoko Wada, Shuichi Murakami, Masaaki Nakano, Ichiei Narita

**Affiliations:** 1 Division of Clinical Nephrology and Rheumatology, Niigata University Graduate School of Medical and Dental Sciences, 1-757 Asahimachi-Dori, Chuo-ku, Niigata City, 951-8510, Japan; 2 Department of Health and Nutrition, Faculty of Human Life Studies, University of Niigata Prefecture, 471 Ebigase, Higashi-ku, Niigata, 950-8680, Japan; 3 Department of Medical Technology, School of Health Sciences, Faculty of Medicine, Niigata University, 2-746 Asahimachi-Dori, Chuo-ku, Niigata City, 951-8518, Japan

**Keywords:** AL amyloidosis, Amyloid-positive area, Creatinine clearance, Estimated GFR, Renal function

## Abstract

**Background:**

The kidney is a major target organ for systemic amyloidosis that often affects the kidney including proteinura, and elevated serum creatinine (Cr). The correlation between amount of amyloid deposits and clinical parameters is not known. The aim of this study was to clarify correlation the amyloid area in all renal biopsy specimen and clinical parameters.

**Methods:**

Fifty-eight patients with an established diagnosis of AL amyloidosis participated in the study. All patients showed amyloid deposits in renal biopsies. We retrospectively investigated the correlation between clinical data and amyloid occupied area in whole renal biopsy specimens.

**Results:**

The area occupied by amyloid was less than 10% in 57 of the 58 patients, and was under 2% in 40. For statistical analyses, %amyloid-positive areas were transformed to common logarithmic values (Log_10_%amyloid). Cr showed significant correlation with Log_10_%amyloid and estimated glomerular filtration rate (eGFR) showed the significant negative correlation. Patient age, cleatinine clearance (Ccr), blood urea nitorogen, and urinary protein was not significantly correlated with Log_10_%amyloid. The correlation with other clinical factors such as sex, and serum concentrations of total protein, albumin, immunoglobulins, compliments was evaluated. None of these factors significantly correlated with Log_10_%amyloid. According to sex- and age- adjusted multiple linear regression analysis, Log_10_%amyloid had significant positive association with Cr and significant negative association with eGFR.

**Conclusion:**

There is significant association between amyloid-positive area in renal tissue and renal function, especially Cr and eGFR. The level of Cr and eGFR may be a marker of amount of amyloid in renal tissue.

## Background

AL amyloidosis is a severe systematic disorder that often affects a systemic symptoms or signs [1]. Amyloid deposition is believed to occur insidiously with age, if patients have had those precursor proteins for long time. Small amounts of amyloid deposition have no ill effects on any organ function, while excessive deposition of amyloid into systemic organs can be potentially fatal [2]. The clinical features of amyloidosis vary by the organ affected. Cardiac amyloidosis causes heart failure [3]. Gut involvement causes disturbances of intestinal movement developing to malabsorption, perforation, hemorrhage, and obstruction [4]. Amyloid deposition into peripheral and autonomic nerves reveals sensory disturbance and orthostatichypotension [4]. Carpal tunnel syndrome is often induced by amyloid deposition into tendons in the wrist joint and is a sensitive clue for systemic amyloidosis when it is bilateral [5]. Skin involvement takes the form of papules, nodules, and plaques. Cutaneous amyloidosis can be a risk factor for non-healing ulcer in elderly persons [6]. The kidney is a major target organ for systemic amyloidosis that often affects the kidney including proteinura, and elevated serum creatinine (Cr). Nephrotic syndrome is present in more than one fourth of patients at the time of diagnosis [7]. The prognosis of renal amyloidosis is generally poor, in AL amyloidosis, mainly because conventional treatments with the previous agents were unable to stop the decline of renal function [8-10]. Many patients will develop end-stage renal failure (ESRF). Additionally, it is generally considered that the quality of life under dialysis is poor with a high rate of mortality [11].

In renal biopsy specimen, histopathologic classification, scoring and grading system for renal amyloidosis was proposed [12]. However, the correlation of these scoring and grading and clinical parameters such as urinary protein or serum creatinine was not fully investigated yet. No clear relationship between the amyloid deposition evident in renal biopsy specimens and the severity of clinical manifestations has yet been demonstrated [12]. Furthermore, the correlation between the amount of amyloid deposits and clinical parameters is not known. In the present study, we investigated the clinical utility of the area of renal biopsy specimens occupied by amyloid and its correlations with various clinical parameters.

## Methods

### Patients and diagnosis of reactive AL amyloidosis

Fifty eight patients with an established diagnosis of AL amyloidosis participated in the study between January 1981 and December 2009. In all patients, the diagnosis was based on renal biopsy, in which the deposition of renal tissue of AL-type amyloid fibril was determined by Congo-red staining on light-microscopic examination. The study protocol was approved by the Institutional Review Board of Niigata University Hospital. We obtained informed consent from all the patients for renal biopsy and to use acquired data. The renal biopsies were performed under ultrasoundguided needle biopsy. The specimens were fixed in 10% phosphate-buffered formalin (pH 7.2), embedded in paraffin, and cut into 4-μm sections. The sections were stained with hematoxylin and eosin, periodic acid Schiff, silver methenamine, and masson trichrome stains for light microscopy to evaluate the glomerular, interstitial, and vascular changes. Congo-red staining of renal tissue specimens was performed for histopathological diagnosis, and green birefringence was considered indicative of the presence of amyloid deposits. We studied the staining pattern of all the samples by polarization light microscopy after treatment of KMnO_4_ to determine the type of amyloidosis. The subtyping of amyloidosis into AL and AA (serum amyloid A) amyloidosis was done by immunofluorescence staining of κ and λ light chains and immunoperoxidase staining for serum amyloid A.

Clinical data were assessed by patient record at the time of renal biopsy. Laboratory index and clinical evaluation of disease activity included determinations of Cr, 24-h proteinuria, 24-h creatinine clearance rate (Ccr), and C-reactive protein (CRP). Other clinical variables, such as total protein, albumin, blood urea nitrogen (BUN), uric acid (UA), and immunoglobulins were assessed by routine laboratory method. Ccr was corrected to body surface area of 1.73 m^2^. Estimated glomerular filtration rate (eGFR) was estimated by the formula described previously [13]. Patients over 80 years of age, or with tubulointerstitial nephritis, were excluded from this study.

### Image analysis of amyloid-positive areas

Renal biopsy specimens were fixed in 10% formalin, embedded in paraffin, and cut into sections 4 μm thick. Sections were considered suitable for quantitative analysis. The amyloid-positive area in the renal tissue was determined on Congo-red-stained sections. One section of whole renal tissue was photographed. The borders of the amyloid-positive areas in each renal tissue section were traced in each photograph, excluding the tissue-free spaces. The total amyloid-positive area was measured with ImageJ v.1.45 software ( http://rsb.info.nih.gov/ij), and the average percentage of the amyloid-positive area per whole-tissue section was calculated. The process of image analysis was previously described [14].

### Statistical analysis

**Figure 1:**
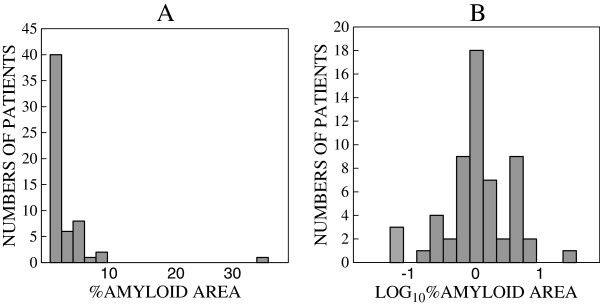
**Histogram showing distribution between numbers of patients and %amyloid area.A**: Distribution between numbers of patients and %amyloid area. Fifty-seven out of 58 patients of %amyloid area were under 10%. Forty out of 58 patients were under 2%. **B**: Distribution between numbers of patients and log_10_%amyloid area. The distribution was similar to normal probability distribution.

For statistical analyses, %amyloid-positive areas were transformed to common logarithmic values (Log_10_%amyloid) since the histograms showed log-normal distribution (Figure 1). Crude correlation between Log_10_%amyloid and each clinical factor were tested using a Pearson’s correlation coefficient. Furthermore, multiple linear regression analysis was applied to assess the sex- and age-adjusted effect of Log_10_%amyloid on each clinical factor, and correlations between medication and Log_10_%amyloid were analyzed using Student’s *t* test. All statistical analyses were performed with SPSS ver. 13 for Windows (SPSS Inc, Chicago, IL, USA) and a *p* value <0.05 was considered statistically significant.

## Results

### Clinical features at the time of biopsy

Sixty patients with renal AL amyloidosis were evaluated in this study. Thirty-five of the patients were male and 23 were female. All of these patients had both symptomatic and asymptomatic signs for amyloidosis. Table 1 shows the clinical characteristics and laboratory findings of these patients at the time diagnosis of amyloidosis was assessed. None of our patients had hypotension. Low levels of serum albumin, abnormal UA, Ccr and proteinuria were also frequent due to renal disorder. Complete monoclonal amyloid light chain was detected in the serum or urine of 52 patients (2 kappa/50 lambda). In the other 6 patients, monoclonal amyloid chain was not detected at the time of renal biopsy. The histological diagnosis of amyloidosis was performed by examination of renal biopsies. Table 2 shows the medication used for treating our patients. Fourteen of them received diuretics. Among the 10 with hypertension, 7 received anti-hypertensive therapy (angiotensin receptor blocker (ARB) in 2, angiotensin converting enzyme inhibitor (ACE) in 1, both ARB and ACE in 1, calcium channel blocker (CCB) in 2). One patient was treated with non-steroidal anti-inflammatory drugs (NSAIDs). Amyloidosis was treated with steroid before chemotherapy. However, these medications were used with the utmost care, while performing frequent renal function tests in order to avoid any adverse effects on renal function, structure or prognosis. Correlations between treatments and Log_10_%amyloid are shown in Table 3. Treatments with diuretics, ACE, ARB, and steroid were not significantly correlated with Log_10_%amyloid.

**Table 1:** Clinical characteristics of patients enrolled in this study

**Characteristic**	**Value**
Male/female, n	35/23
Mean age at renal biopsy, yrs (SD) [range]	62.7 (12.9) [20–87]
Systolic blood pressure (mmHg)	118.6 (18.2) [146–80]
Diastolic blood pressure (mmHg)	72.0 (10.4) [94–50]
Total protein (g/dl)	5.0 (0.8) [3.6–6.9]
Albumin (g/dl)	2.5 (0.7) [1.0–4.4]
BUN (mg/dl)	17.0 (8.4) [7.2–62.9]
Serum creatinine (mg/dl)	0.9 (0.4) [0.4–1.9]
Uric acid (mg/dl)	5.8 (1.8) [1.6–10.6]
Na (mEq/L)	140.1 (3.1) [132–145]
K (mEq/L)	4.1 (0.4) [3.2–5.1]
Ca(mg/dl)	8.2 (1.2) [4.0–9.6]
P(mg/dl)	4.4 (2.5) [2.8–4.5]
ALT(IU/ml)	30.7 (15.8) [12–79]
AST(IU/ml)	32.0 (41.8) [7–248]
ALP(IU/ml)	256.2 (204.8) [50–797]
LDH(IU/ml)	348.7 (168.3) [167–778]
Total bilirubin (mg/dl)	0.5 (0.3) [0.2–1.1]
Total cholesterol (mg/dl)	280.8 (91.0) [158–597]
Triglyceride (mg/dl)	176.3 (113.4) [66–583]
Creatinine clearance (ml/min/1.73 m2)	75.5 (34.9) [20.2–214.0]
Urinary protein (g/day)	4.0 (3.9) [0.3–29]
Immunoglobulin G (mg/dl)	875.5 (537.1) [110–3061]
Immunoglobulin A (mg/dl)	265.1 (285.5) [30–1250]
Immunoglobulin M (mg/dl) M-protein (κ/λ/N.S.)	102.1 (34.1) [14–225] 2/50/6
C3 (mg/dl)	103.2 (61.2) [14–225]
C4 (mg/dl)	35.2 (13.6) [12.0–83.4]
CH50 (U/ml)	39.9 (11.2) [5–65]
eGFR (ml/min/1.73 m^2^)	79.5 (32.9) [21.0–134.7]

*SD*, standard deviation; *BUN*, Blood urea nitrogen; *CH50*, 50% hemolytic unit of complement; *eGFR*, estimated glomerular filtration rate; *NS*, not specific.

**Table 2:** Therapy of the patients at the time of renal biopsy

**Therapy**	**Number of patients (percent)**
No therapy	29 (50.0)
Diuretics	14 (24.1)
Frusemide	12 (20.7)
Frusemide + Spironolactone	1 (1.7)
Fluitran	1 (1.7)
Steroid	6 (10.3)
ARB	3 (4.4)
ACE	1 (1.7)
ARB + ACE	1 (1.7)
CCB	2 (3.4)
NSAIDs	1(1.7)
Unkown	1(1.7)

*ARB*, Angiotensin receptor blocker; *ACE*, Angiotensin converting enzyme inhibitor; *CCB*, calcium channel blocker; *NSAIDs*, Non-steroidal anti-rheumatoid drugs.

**Table 3:** **Correlation between treatment and log**_**10**_**%amyloid**

	**Treated**			**Non-treated**			**p-value**
	**n**	**Mean**	**±SD**	**n**	**Mean**	**±SD**	
Treatment	28	0.039	±0.616	30	0.051	±0.551	0.935
Diuretics	14	−0.065	±0.609	44	0.080	±0.570	0.418
ACE	2	0.178	±0.662	56	0.041	±0.581	0.745
ARB	4	0.449	±0.297	54	0.015	±0.584	0.149
Steroid	5	0.190	±0.663	53	0.032	±0.574	0.935
NSAID	1	−1.347		57	0.070	±0.552	

### Renal histological findings

We evaluated areas of tissue that contained an average of 17.9 glomeruli and showed an area of global sclerosis (GS) of about 1.65 glomeruli on average. Glomerular damage without amyloidosis was not detected 57 of the 58 patients. Only one patient showed mild focal mesangial proliferation by light microscopy. Figure 1A shows a histogram of the distribution of the numbers of patients and %amyloid area. The %amyloid area was <10% in 57 of the 58 patients, and was <2% in 40. Figure 1B showed distribution between numbers of patients and log_10_%amyloid area. The distribution was similar to normal probability distribution.

**Figure 2:**
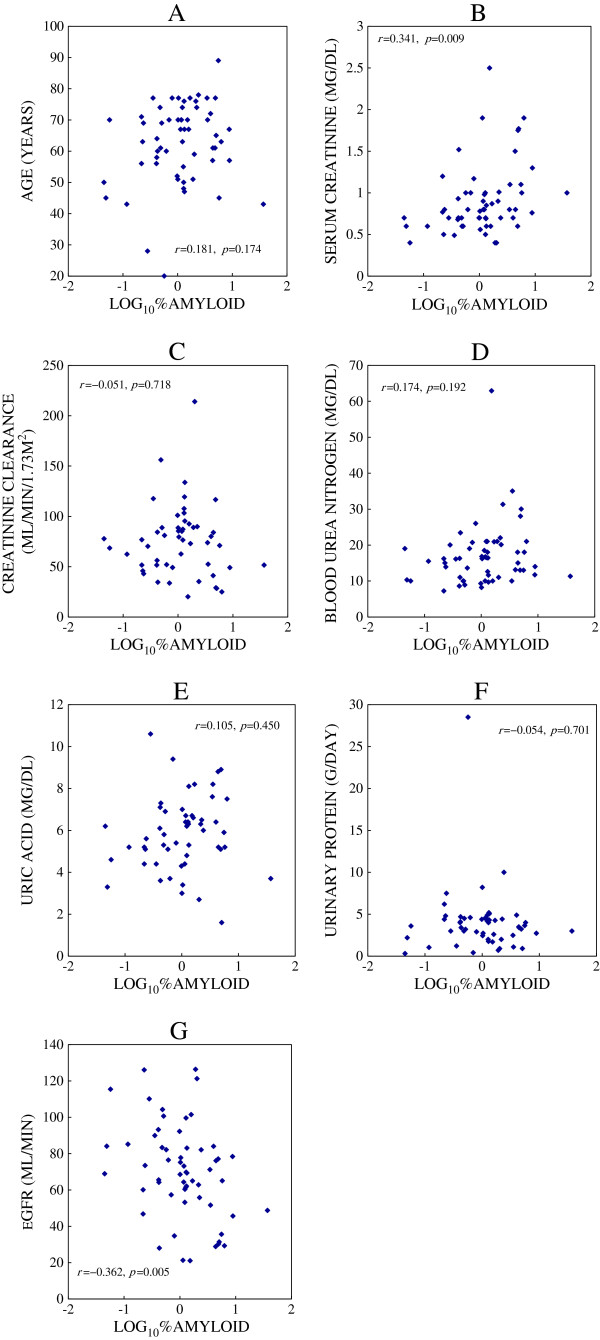
**Correlation between Log**_**10 **_**%amyloid and variables. A**: Correlation between Log_10_%amyloid and age. **B**: Correlation between Log_10_%amyloid and serum creatinine. **C**: Correlation between Log_10_%amyloid and creatinine clearance. **D**: Correlation between Log_10_%amyloid and blood urea nitrogen. **E**: Correlation between Log_10_%amyloid and uric acid. **F**: Correlation between Log_10_%amyloid and urinary protein. **G**: Correlation between Log_10_%amyloid and eGFR. Patient age was significantly correlated with Log_10_%amyloid. Cr and eGFR showed significant correlation with Log_10_%amyloid. Other clinical parameters did not showed significant correlation with Log_10_%amyloid.

The correlation between Log_10_%amyloid and selected clinical factors were shown in Figure 2. Cr (*r* = 0.341, *p =* 0.009, Figure 2B) showed significant correlation with Log_10_%amyloid and eGFR (*r* = −0.362, *p =* 0.005, Figure 2G) showed the significant negative correlation. Patient age (*r* = 0.181, *p* = 0.174, Figure 2A), Ccr (*r* = −0.051, p = 0.718, Figure 2C), BUN (*r* = 0.174, *p* = 0.192, Figure 2D), UA (*r* = 0.105, *p* = 0.450, Figure 2E) and urinary protein (*r* = −0.054, *p* = 0.701, Figure 2F) was not significantly correlated with Log_10_%amyloid. We also analyzed the correlation with other clinical factors such as sex, and serum concentrations of sodium, potassium, total protein, albumin, immunoglobulins (immunoglobulin G (IgG), immunoglobulin A (IgA), and immunoglobulin M (IgM)), compliments (C3, C4), and total hemolytic component (CH50). None of these factors significantly correlated with Log_10_%amyloid.

According to sex- and age- adjusted multiple linear regression analyses, Log_10_%amyloid had significant positive association with Cr and significant negative association with eGFR as shown in crude correlation analyses (Table 4). Furthermore, Log_10_%amyloid did not appeared to have significant positive association with Ccr, BUN, UA, and urinary protein when the effects of sex and age were adjusted.

**Table 4:** **Sex- and age-adjusted association between log**_**10**_**%amyloid and each clinical variable**

	**Regression coefficient (95% CI)**		***p*-value**
Serum creatinine (mg/dL)	0.22	(0.03–0.40)	0.024
Creatinine clearance	0.43	(-16.7–17.5)	0.961
Serum urinary nitrogen (mg/dL)	1.81	(-2.13–5.75)	0.373
Serum uric acid (mg/dL)	0.12	(-0.70–0.94)	0.778
Urinary protein (g/day)	-0.17	(-1.95–1.61)	0.849
eGFR (ml/min/1.73 m^2^)	-15.2	(-26.3–-4.2)	0.009

*EGFR*, estimated glomerular filtration rate.

Regression coefficient adjusted for sex and age by multiple linear regression analysis.

## Discussion

AL amyloidosis can be classified as either primary amyloidosis or secondary to multiple myeloma, on the basis of the number of plasma cells in the bone marrow and/or the presence or absence of skeletal lesions that frequently involve the kidneys [15]. Once the symptoms of amyloidosis have manifested, disease progression is usually rapid [14]. Kyle, et al. reported that, based on a multi-variate analysis of 168 AL amyloidosis patients, renal function was a predictor of survival 1 year after diagnosis [15]. Other study demonstrated that the long-term prognosis of renal amyloidosis is significantly poorer if Cr concentration at the time of biopsy is 1.3 mg/dl or more [16]. Additionally the progression of renal pathological damage due to amyloid deposition expected a short renal survival time and poor individual survival [16]. In a study of 63 AL amyloidosis patients with renal amyloidosis found that high Cr concentration at biopsy and interstitial and/or vascular damage by amyloid deposition accelerated the progression of renal dysfunction [17]. The findings of the study confirmed that both renal function and interstitial damage at biopsy were predictive factors for prognosis of survival, but mesangial expansion was not [18]. In AL amyloidosis, renal amyloid deposits were frequently deposited in order. Amyloid deposits were initially deposited inside the glomrular basement membrane and mesangium is a principal site of amyloid deposition in glomeruli. Amyloid deposits may occur in segmental, diffuse mesangial, nodular, and pure basement membrane pattern [19]. Segmental amyloid deposits are small, discrete, and confirmed to the masangium without forming nodules. Although this form is so small but it is pointed out that these small deposits can be associated with massive proteinuria. In our study, these small nodules were included in the amyloid positive area, although these small nodules might be the reason of massive proteinura. This might be the reason that Log_10_%amyloid was not correlated with urinary protein. In general, the tubular interstitial component may be variably affected. The tubules may show nonspecific findings or vacuolization and damage. Interstitial and peritubular deposits of amyloid are seen in approximately 50% of cases. Medullary amyloid deposits are more frequent and more extensive [20].

In renal vessels, they are often involved, with arteriolar deposits being most frequent, followed by deposits in arteries, peritubular capillaries and veins. Vascular deposits frequently coexist with glomerular amyloid, but the extent of vascular amyloid deposition is unrelated to the pattern of glomerular involvement [21]. These morphological courses of renal depositions are considered the progression of renal involvement may reflect the clinical parameters.

Our results showed the significant correlation between Log_10_%amyloid and eGFR, but Log_10_%amyloid was not correlated with Ccr, because the dissociation was existed between eGFR and Ccr. The international standard method to measure glomerular filtration rate (GFR) is inulin clearance (Cin). However, Cin was not applied in clinical practice in Japan. Ccr is frequently made use of measurement of renal function, because of its simple method. The dissociation between Ccr and GFR is known in previous studies [22]. Usually Ccr shows higher than the value of GFR. In Japanese, approximately the revel of Ccr was 20% higher than the level of Cin [23]. In our results of Table 1 showed the dissociation between Ccr and eGFR. Ccr is about 8% higher that eGFR. Therefore, eGFR is considered to be closer to the GFR level than Ccr. Log_10_%amyloid may correlate as true renal function of GFR. Recently, we revealed that there was a significant correlation between the area of amyloid deposition in renal tissue and parameters of renal function in AA amyloidosis associated with rheumatoid arthritis and the results were similar to this study [14]. In AA amyloidosis, we also revealed that the treatment of biologics for rheumatoid arthritis reduce the amyloid deposits in gastroduodenal biopsy specimen [24]. Some cases in these AA amyloidosis patients reduce Cr and elevated Ccr were observed for these patients. In AL amyloidosis, specific therapy was significantly prolongs survival and slows the progression of renal disease [25]. Repeated biopsy of kidney was difficult in practice but fluctuation of Cr of eGFR may reflect the amount of deposition of renal tissue in AL amyloidosis patients. Recently, the serum free light chain (sFLC) concentration has been shown to be a reliable predictor of the response and progression of multiple myeloma. However, only one-third of the patients in our study underwent sFLCs measurement, and because of the large number of missing data, we did not include this parameter in the study.

## Conclusion

In conclusion, there is significant association between amyloid positive area in renal tissue and renal function, especially Cr and eGFR. Urinary protein and Ccr was an important marker in renal amyloidosis, but not correlated with amyloid-positive area. Medication with diuretics, ACE, ARB or steroid at the time of renal biopsy was not correlated with the area of amyloid positivity in renal tissue. The level of Cr and eGFR may be a marker of the amount of amyloid in renal tissue. As this was a cross-sectional study, further follow-up of patients for a longer time to observe the progression of CKD in relation to the area of amyloid positivity will be necessary to confirm our observations.

## Competing interests

None of the authors has a conflict of interest to declare.

## Authors’ contributions

TK, DK, YW and SM carried out the data acquisition. TK and NT did the data administration. TK, NT and MN performed statistical analysis. TK, MN and IN wrote the manuscript. All authors read and approved the final manuscript.

## Pre-publication history

The pre-publication history for this paper can be accessed here:

http://www.biomedcentral.com/1471-2369/13/118/prepub
